# Sequelae of Acute Respiratory Distress Syndrome: Interest of Rehabilitation

**DOI:** 10.1155/2019/7953141

**Published:** 2019-03-06

**Authors:** Elise Godeau, David Debeaumont, Elise Artaud-Macari, Laurie Lagache, Gurvan Le Bouar, Jérémy Coquart

**Affiliations:** ^1^CHU-Hôpitaux de Rouen, Unité de Physiologie Respiratoire et Sportive, 76000 Rouen, France; ^2^CHU-Hôpitaux de Rouen, Service de Pneumologie, Oncologie Thoracique et Soins Intensifs Respiratoires, 76000 Rouen, France; ^3^Normandie Univ, UNIROUEN, Institut de Recherche et d'Innovation Biomédicale, EA3830: Groupe de Recherche sur le Handicap Ventilatoire, 76000 Rouen, France; ^4^CHU-Hôpitaux de Rouen, Service de Réanimation Médicale, 76000 Rouen, France; ^5^Normandie Université, UNIROUEN, EA 3832: Centre d'Etudes des Transformations par les Activités Physiques et Sportives, Institut de Recherche Interdisciplinaire Homme et Société, 76130 Mont-Saint-Aignan, France

## Abstract

**Case Presentation:**

This clinical case presents the history of a woman hospitalized for acute respiratory distress syndrome (ARDS). A 62-year-old woman, with regular physical activity and no history of respiratory disease or smoking, was hospitalized for moderate ARDS with bilateral pneumonitis. Fourteen days later, she was discharged from the intensive care unit and received respiratory physical therapy. One month later, she experienced exertional dyspnea. A regression of alveolar condensation with persistent sequelae at the pulmonary bases was noted. Three months later, the patient continued daily physical activity with satisfactory tolerance. A reduction in alveolar-capillary transfer, inappropriate hyperventilation upon exercise, and impairment of gas exchanges at maximal effort, suggestive of pulmonary shunt, were demonstrated. At the 6-month evaluation, the patient displayed exertional dyspnea with residual bilateral basal consolidations. Six months later, the dyspnea had ceased. The persistence of bilateral basal interstitial syndrome associated with bronchial dilatation and pleural-based consolidations was noted, as well as a stable impaired alveolar-capillary diffusing capacity.

**Discussion:**

Upon discharge from intensive care, pulmonary follow-up should be proposed to ARDS survivors. Moreover, pulmonary function testing at rest and exercise is advised as soon as possible to evaluate the respiratory sequelae. This will help to limit the severity of complications through adapted exercise rehabilitation and then regular physical activity.

## 1. Introduction

Acute respiratory distress syndrome (ARDS) accounts for 10.4% of intensive care admissions and is associated with high mortality, between 34.9 and 46.1% depending on the severity [1]. ARDS is a multifactorial syndrome characterized by (i) new or worsening symptoms during 1 week, (ii) bilateral opacities (not fully explained by effusions, lobar/lung collapse, or nodules), (iii) the ratio of arterial oxygen tension to fraction of inspired oxygen less than 300 mm Hg with positive end-expiratory pressure or continuous positive airway pressure of 5 cm H_2_O or more, and (iv) no clinical evidence of heart failure or fluid overload [2]. It causes physiological alterations: alteration of pulmonary thoracic compliance, intra-lung shunt, and alveolar derecruitment responsible for severe hypoxemia, which can be generated or aggravated by invasive mechanical ventilation [3]. The causes of ARDS can be respiratory (infectious or inhalation pneumonitis) or extra respiratory (acute pancreatitis). In addition to etiological treatment, the implementation of a specific ventilation system with a low tidal volume (called protective) is essential for the management of ARDS [4]. The main complication is the progression to chronic pulmonary fibrosis.

Long-term sequelae are frequently observed in survivors, with physical and/or psychological impairments and altered higher functions that directly reduce the quality of life [5]. In addition, the impaired lung function may decrease exercise tolerance [6, 7]. Although the sequelae may diminish over time, they rarely disappear, even after 5 years [7]. Monitoring of lung function after ARDS is currently not systematic, and sequelae are therefore often detected somewhat late. Yet early assessment of impaired lung function, especially at exercise, would help to optimize management by preventing or limiting the severity of complications and ultimately reducing health costs.

## 2. Case Presentation

A 62-year-old woman (body mass: 61.2 kg; height: 1.57 m; body mass index (BMI): 24.8 kg.m^−2^) was hospitalized in an intensive care unit for ARDS of infectious origin.

Her medical history included autoimmune hypothyroidism, arterial hypertension, and anosmia. She reported no history of smoking. She was retired and practiced regular physical activity: walking, hiking, and using a home stepper. She was only treated by levothyroxine.

**Figure 1:**
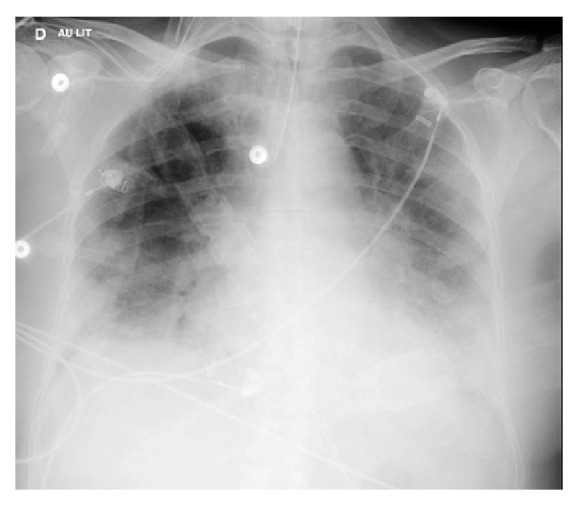
The chest X-ray (in supine position) on admission showing bilateral alveolar-interstitial syndrome with bilateral lower lobe consolidations.

The patient was first seen as an outpatient with influenza-like symptoms and was treated with probabilistic antibiotic therapy (amoxicillin then ceftriaxone) for 7 days. The evolution was unfavorable and she was admitted to the pulmonary critical care unit on 5 April 2016 with signs of acute respiratory distress. The chest X-ray on admission showed bilateral alveolar-interstitial syndrome with bilateral lower lobe consolidations (Figure 1), prompting spiramycin addition to her antibiotic treatment. Following a rapid deterioration in lung function under high-flow oxygen therapy, the patient was intubated (D+1) and transferred to the intensive care unit. The worsening clinical picture prompted antiviral treatment with oseltamivir (75 mg twice daily) in addition to ceftriaxone (2 g) and spiramycin (1.5 million units daily). Repeated samples were taken for bacteriological (i.e., protected brush sampling of mucus plugs, cytobacteriological examination of urine, blood culture, polymerase chain reaction, and antigenuria) and virological (e.g., polymerase chain reaction for influenza) investigations. The patient was treated with norepinephrine up to 0.5 microgramme.kg^−1^.min^−1^ for 24 hours because of hemodynamic instability.

Moderate ARDS (partial pressure arterial oxygen/fraction of inspired oxygen ratio = 123 mmHg with positive end-expiratory pressure ≥ 5 cm H_2_O) with bilateral pneumonitis was diagnosed.

The patient was placed on mechanical ventilation at 6 mL.kg^−1^ predicted body weight, along with sedation using midazolam and sufentanil and curarization with cisatracurium for 48 hours. The favorable response led to a gradual decrease in sedation and ventilatory weaning at D+10. However, following respiratory distress secondary to laryngeal edema, the patient was reintubated, and systemic corticosteroid therapy (1 mg.kg^−1^) was initiated. In the absence of bacteriological evidence and given the favorable clinical course, antibiotic therapy was reduced to ceftriaxone alone for 10 days. The definitive extubation (D+13) proceeded without complication. The patient (body mass: 62.0 kg, i.e., BMI: 25.2 kg.m^−2^) was discharged from the intensive care unit (D+14) after weaning from oxygen. This delay is according to the literature, which recommends between 2 and 3 weeks [8]. She then received respiratory physical therapy in the after-care and rehabilitation department.

The rehabilitation program comprised physical exercises [9]. The endurance exercises were performed on a cycle ergometer, treadmill, or stepper. Initially, endurance exercises were executed in sequences of 10 minutes or less, with the goal of reaching 30 minutes per session. The intensity of endurance exercises was prescribed on the effort perception (score of 11-13 on the ratings of perceived exertion scale) [10]. In addition, muscle strengthening exercises for the upper and lower limbs were proposed, lasting 10-15 minutes per session and using weights, dumbbells, or elastic bands. Each exercise comprised a series of 6-12 repeated movements. A 1-minute recovery period was observed between exercises. Warm-up and stretching exercises were performed, respectively, before and after each session [10]. Moreover, the patient was encouraged to increase the time spent in daily living activities.

**Figure 2:**
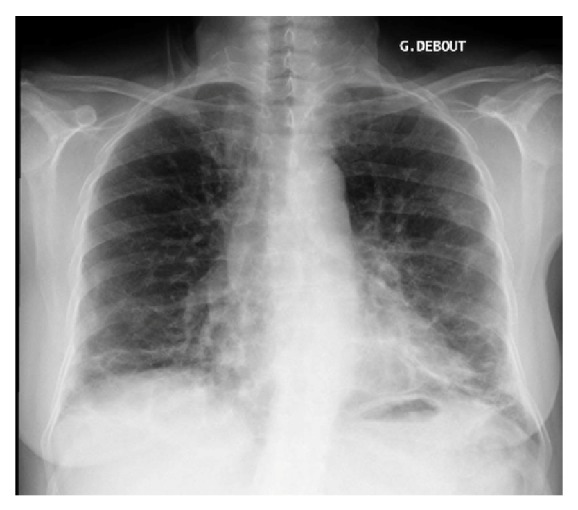
The chest X-ray (in standing position) showing the clear regression of alveolar condensation with some persistent sequelae at both pulmonary bases 1 month after ARDS.

One month after this episode, she experienced dyspnea on exertion with whitish sputum and a 2-kg weight gain despite daily physical activity (30 minutes/day on a stepper). At D+39, a new chest X-ray showed the clear regression of alveolar condensation with some persistent sequelae at both pulmonary bases (Figure 2).

Three months later, the patient (body mass: 63.0 kg, i.e., BMI: 25.6 kg.m^−2^) was seen in consultation in the pulmonary department. She had continued daily physical activity with satisfactory tolerance (30 minutes/day of treadmill walking and 15 minutes/day cycling at moderate intensity). Pulmonary function testing (PFT) revealed impaired alveolar-capillary transfer with a carbon monoxide diffusing capacity reduced to 3.93 mmol/(min*∗*kPa) (60% of the theoretical value). The pulmonary volumes and expiratory flow rates were normal (forced expiratory volume second = 1.84 L, vital capacity = 2.2 L, total lung capacity = 4.49 L; i.e., 102%, 98%, and 109% of the respective theoretical values), with no sign of obstruction (Tiffeneau ratio: 87.1%). Respiratory muscle strength was normal (based on maximal inspiratory and expiratory pressures and the sniff test). The patient covered a satisfactory distance of 542 m (i.e., 109% of the theoretical value) [11] in the 6-minute walk test, with significant desaturation (i.e., 8% drop in saturation, with 88% saturation at the end of the test). The cardiopulmonary exercise test indicated normal aerobic fitness with a peak oxygen flow (VO_2peak_) of 20.4 mL.min^−1^.kg^−1^. The exercise intolerance appeared to be ventilatory in nature with low ventilatory reserve (RV = 6.3%), high respiratory quotient (RQ = 1.32), inappropriate hyperventilation (69 L.min^−1^) with high respiratory equivalents, significant desaturation on exertion, and a high alveolar-arterial gradient (8.1 kPa), consistent with ARDS sequelae. The exercise limitation also appeared to be of metabolic origin, related to overweight. She was advised to continue regular physical activity (at the first ventilatory threshold) at home, where she had a treadmill and an ergocycle.

At the 6-month evaluation, the patient displayed only mild exertional dyspnea, stage 1 of the modified Medical Research Council (mMRC) classification, but her physical activity was limited by pain from a sternal fracture caused by a road accident (August 2016). The chest CT scan confirmed residual bilateral basal consolidations.

**Figure 3:**
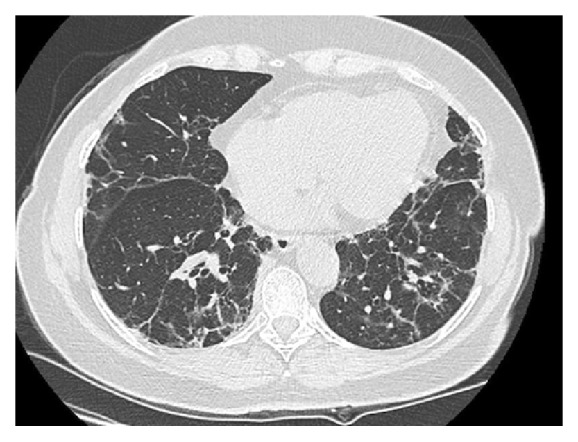
The chest CT scan showing the persistence of a bilateral interstitial syndrome associated with bronchial dilatation and pleural-based consolidations 1 year after ARDS.

At one year, the patient no longer experienced dyspnea. The chest CT scan showed the persistence of a bilateral interstitial syndrome associated with bronchial dilatation and pleural-based consolidations (Figure 3). PFT had remained stable (carbon monoxide diffusing capacity: 63%) and no other abnormality could be detected.

## 3. Discussion

ARDS is an acute edema of the lungs with a high mortality rate. For this reason, it usually requires admission to an intensive care unit. The progression of histological lesions has been described as resulting in diffuse alveolar damage. During the acute exudative phase, an inflammatory process in the pulmonary alveoli causes the diffuse alveolar damage and generates disturbances in the alveolar-capillary permeability. During the fibroproliferative phase (between D+7 and D+14), a large amount of collagen is secreted by type II pneumocytes, causing remodeling of the interstitium and the alveolar spaces. The chronic phase is characterized by either pulmonary repair or the progression to fibrosis. It should be noted that these phases overlap [12], and the specificity of the syndrome is the loss of a compartmentalized inflammation response with the perpetuation of pulmonary aggression even when the initiating phenomenon has been controlled.

The respiratory sequelae in ARDS are related to alveolar-capillary membrane impairment with diminished exercise capacity, which affects patient independence and quality of life, especially in the year following diagnosis [13]. These sequelae diminish over time but do not disappear, even over the long term. Herridge et al. [7] followed patients hospitalized for ARDS for 5 years. At each consultation (3 in the first year and once a year thereafter), the patients were clinically examined, underwent a chest X-ray, PFT, a 6-minute walk test, and exercise oximetry, and responded to a quality of life questionnaire. The results indicated significantly low exercise tolerance with little distance covered during the walking test, reduced vital capacity, diffusion limitation in alveolar-capillary transfer capacity, and low quality of life scores. The physical and psychological sequelae persisted at 5 years, with a significant impact on the health-related quality of life. Usually, this long-term substantial reduction in health-related quality of life in surviving ARDS does not differ from findings in patients surviving other critical illness [14]. In addition to these adverse consequences for the patient, ARDS represents a significant public health cost and early management strategies are thus needed.

Moreover, it has been reported that a stay in the intensive care unit has functional consequences for ARDS patients, notably muscle weakness [15]. The literature therefore recommends early exercise rehabilitation, and we encouraged our patient to return to her regular physical activity [16].

Indeed, thanks to exercise rehabilitation and then physical exercises at home, the patient no longer experienced dyspnea, and she indicated to have an acceptable quality of life and to be able to execute the activities of daily living. Thus, cardiopulmonary exercise test has not been repeated. However, it would have been interesting to accurately evaluate exercise tolerance from field tests. Field tests are low-cost, require little equipment, and are considered to be more reflective of daily living than cardiopulmonary exercise test [9]. In numerous respiratory diseases [9] and especially in ARDS [17], the most established is the 6-minute walk test. Nevertheless, it must be performed in an unobstructed 30-metre hallway [18], and such a space is rarely available in the patient's home, in the physician's office, or even in some respiratory medicine departments. Thus, to overcome this spatial limitation, a 6-minute stepper test has recently been proposed [19]. This exercise test requires only a limited amount of space and equipment and is feasible, valid, reproducible, and sensitive in patients with respiratory diseases [19, 20]. Consequently, this field exercise test might be recommended to evaluate tolerance exercise in ARDS survivors, when cardiopulmonary exercise test or 6-minute walk test is unavailable. Nevertheless, further studies are needed.

The respiratory sequelae of ARDS had a significant clinical impact on our patient, especially during exercise. These sequelae were confirmed by the pulmonary evaluations at 3, 6, and 12 months. Moreover, overweight might have had an impact on the clinical response to exercise. Finally, the pain from a sternal fracture at the 6 months has limited patient's physical activity, and thus it is possible that, without the road accident, the benefits would have been better.

As was the case for our patient and in the studies cited, post-intensive care management should be systematic for ARDS survivors, with early pulmonary evaluation at exercise (3 months) and, when possible, functional and respiratory rehabilitation to improve the respiratory function prognosis.

## 4. Conclusion

Upon discharge from intensive care, pulmonary follow-up should be systematically proposed to ARDS survivors. In addition, a control chest X-ray and pulmonary function testing at rest and exercise are advised as soon as possible to evaluate the respiratory sequelae. This will help to prevent or limit the severity of complications through adapted exercise rehabilitation, which should then be followed by regular physical activity. In this way, short-, medium-, and long-term prognosis are improved, as well as quality of life.
